# Identifying individual animal factors associated with *Mycobacterium avium* subsp. *paratuberculosis* (MAP) milk ELISA positivity in dairy cattle in the Midwest region of the United States

**DOI:** 10.1186/s12917-018-1354-y

**Published:** 2018-01-25

**Authors:** Gustavo Machado, Kaushi Kanankege, Val Schumann, Scott Wells, Andres Perez, Julio Alvarez

**Affiliations:** 1 Department of Veterinary Population Medicine, University of Minnesota, St. Paul, MN USA; 2 Department of Population Health and Pathobiology, College of Veterinary Medicine, North Carolina State University, 1060 William Moore Drive, Raleigh, NC USA; 3 Minnesota DHIA, Minnesota Dairy Herd Improvement Association, Buffalo, MN USA; 4 Raleigh, USA

**Keywords:** Dairy cattle, Mixed-model, *Mycobacterium avium* subsp. *paratuberculosis*, Somatic cell count, ELISA

## Abstract

**Background:**

*Mycobacterium avium* subsp. *paratuberculosis* (MAP) is a widespread chronic disease of ruminants that causes severe economic losses to the dairy cattle industry worldwide. The objective of this study was to evaluate the association between individual cow MAP-ELISA and relevant milk production predictors in dairy cattle using data routinely collected as part of quality and disease control programs in the Midwest region of the U.S. Milk ELISA results of 45,652 animals from 691 herds from November 2014 to August 2016 were analyzed.

**Results:**

The association between epidemiological and production factors and ELISA results for MAP in milk was quantified using four individual-level mixed multivariable logistic regression models that accounted for clustering of animals at the farm level. The four fitted models were one global model for all the animals assessed here, irrespective of age, and one for each of the categories of < 4 year-old, 4–8 year-old, and > 8 year-old cattle, respectively.

A small proportion (4.9%; *n* = 2222) of the 45,652 tested samples were MAP-seropositive. Increasing age of the animals and higher somatic cell count (SCC) were both associated with increased odds for MAP positive test result in the model that included all animals, while milk production, milk protein and days in milk were negatively associated with MAP milk ELISA. Somatic cell count was positively associated with an increased risk in the models fitted for < 4 year-old and 4–8 year-old cattle. Variables describing higher milk production, milk protein content and days in milk were associated with significantly lower risk in the models for 4–8 year-old cattle and for all cattle.

**Conclusions:**

Our results suggest that testing cows with high SCC (> 26 × 1000/ml), low milk production and within the first 60 days of lactation may maximize the odds of detecting seropositive animals. These results could be useful in helping to design better surveillance strategies based in testing of milk.

**Electronic supplementary material:**

The online version of this article (10.1186/s12917-018-1354-y) contains supplementary material, which is available to authorized users.

## Background

*Mycobacterium avium* subsp. *paratuberculosis* (MAP), also known as paratuberculosis, is a chronic, gastrointestinal disease affecting domestic ruminants. MAP is caused by infection with MAP, an agent capable of infecting a wide range of domestic and wild animals, specially ruminants [1]. MAP is an OIE notifiable terrestrial disease (OIE-listed diseases, infections and infestations in force in 2016-http://www.oie.int/animal-health-in-the-world/oie-listed-diseases-2016/), and in the U.S., based on the most recent herd study, herd-level prevalence are as high as 90% [2], indeed MAP is a major problem for livestock health and productivity not only in U.S. but also globally.

The annual economic impact of MAP on the U.S. livestock industry has been estimated to of the order of hundreds of millions of dollars, and losses to the dairy industry alone are estimated to reach $200–250 million [3]. Absence of effective control measures to reduce MAP within-farm prevalence reduces farm income due to increased premature animal removal [4] and costs associated with MAP diagnostic testing [5]. In addition, MAP infection often leads to reduced milk production and quality, such as low fat and protein content [6], premature mortality, weight loss, early culling, and reduced slaughter value [7–9]. However, contradict results regarding the impact of MAP on milk yield and days in milk have been reported. This is especially true when serological tests are used to define MAP-infection status [10–12], and may be related to the long incubation period of the disease. The long incubation period also impairs the ability to use standard definitions for infected animals across studies difficult. Evaluating the impact of MAP infection is further complicated by differences in samples and diagnostic tests used in different studies.

At the individual level, presence of MAP antibodies has been linked to lactation number, lactation persistence (defined as the rate of decline in production after the peak milk production has been reached) [13] and milk production [11, 12, 14]. Age is another individual factor typically associated with increased odds of positivity [15], likely due to the course of MAP infection that is expected to be age- and infection-stage specific [16]. In addition, some found evidence of a seasonal pattern in birth of ELISA-positive cows in US dairy herds [17]. Nevertheless, due to the lack of periodic testing and standardized definitions of what an infected animal is, inconsistent epidemiological findings for MAP are quite common [18].

In the state of Minnesota there are 3470 licensed dairy herds and 460,000 dairy cattle (Minnesota Department of Agriculture- https://www.mda.state.mn.us/food/business/~/media/Files/food/business/economics/dairyindprofile.pdf), and the state ranks 7th in number of cows and 8th in milk production nationally, based on the latest data from the United States Department of Agriculture-National Agriculture Statistics Service (USDA-NASS). In MN, individual MAP prevalence has been estimated via diverse sample sources, ranging from 10% to 14% [19], whereas a within-herd mean of 13% positive animals, alternating from 0% to 67% was estimated based on ELISA [20].

The Dairy Herd Improvement Association (DHIA) stores information concerning milk production and quality, and offers testing for MAP to participant farms. In 2016 the participant farms included 1625 dairy herds in MN. Here, we used an individual-level mixed model that accounted for clustering of tested animals within each farm for the identification of characteristics associated with seropositivity in testing DHIA herds. The objectives of this study were a) to quantify the impact of MAP infection (defined by positive milk-ELISA results) on milk production indicators, and b) to identify cow-level possible risk factors associated with positive milk ELISA results in dairy cattle. These results can help to understand MAP dynamics in dairy cattle herds and design age-specific risk-based MAP management plans.

## Methods

### Study population and data definitions

Information on production and individual characteristics from dairy cattle (Minnesota (93%), Wisconsin (7%), South Dakota (1%), and Iowa (1%)) subjected to MAP testing between November 2014 and August 2016 was provided by the DHIA. Data included 55,298 tests performed on 46,114 cattle from 691 dairy herds. Because a subset of animals (18.21%) was tested more than once during the study period, if all results were negative, only the result from the first sample was retained for analysis. If one or more tests were positive, the first positive result was kept for further analysis. After that correction, we analyzed 46,114 individual observations.

Animals were classified as positive, inconclusive or negative based on a milk ELISA [IDEXX MAP Enzyme Linked Immunosorbent Assay (ELISA) kit (IDEXX Laboratories Inc., Maine, USA)] according to the manufacturer’s instructions.

### Explanatory variables

Variables determined in the same milk sample used for the MAP test that were available to us included sampling date, animal individual identification, date of birth, lactation number, milk production of the cow per day, fat and protein percentage in milk, reduced lactation persistency, milk urea nitrogen values, SCC, days in milk (DIM), and the 305-day mature equivalent milk production (305ME). No individual breed information was available, although overall ~ 90% of the animals in the studied population were Holstein. Age at sampling was categorized into three classes < 4 years, 4-to-8 years, and > 8 years. In addition, season at birth (Winter [December–January-February]; Spring [March–April-May]; Summer [June–July-August] and Fall [September–October-November]) was used in the analysis. Continuous variables were also categorized into quartiles and explored in the analysis to ensure satisfactory statistical power [21].

### Data analysis

Due to the hierarchical structure of the data (i.e. multiple cows from each herd), we performed a logistic regression analysis using a generalized linear mixed model in which the output was the MAP ELISA result and herd was included as a random effect. The outcome variable was test result (positive/negative), and inconclusive results were removed from the database prior to fitting the models. First, all variables were screened in univariable models using a *p*-value ≤0.15 as a liberal threshold for consideration in the multivariable model [22]. Collinearity between pre-selected explanatory variables was then explored using Spearman’s rank correlation coefficients for not normally distributed continuous variables. For variables with a correlation coefficient ≥ |0.50|, only the variable with the highest biological plausibility and/or with the strongest association to the output was maintained in the multivariable analysis.

Two-way biologically plausible interactions between pre-selected explanatory variables were investigated, and maintained in the multivariable selection when the *p*-value of the cross product was ≤0.20. Multivariable models were fit using a manual backward stepwise selection, so that non-significant variables (*p* > 0.05) that were not acting as confounders (a change > 40% in the coefficient of another covariate was observed when removed) or effect modifiers were removed from the multivariable model, until only explanatory variables were retained (*p* ≤ 0.05).

The age and DIM were considered a priori confounders based on previous evidences [11, 23, 24] and forced into all models. In addition, in order to get a better understanding of the effect of the covariates in the different production stages of the animal, three independent multivariable models were also fitted for each of the categories of < 4 year-old (*n* = 26,357); 4–8 year old (*n* = 18,626) and > 8 year old (*n* = 1131), as described above.

Estimated odds ratios (OR) and 95% confidence intervals were obtained as measures of predictor effect. Model fit was assessed using the deviance, and significant (Mann-Whitney test) difference in the predicted probability of positive result between observed positive and negative observations was considered evidence of adequate goodness-of-fit. The predictive capacity of the final model by estimating the area under the receiver operating characteristic (ROC) curve analysis with 95% probability intervals computed using 2000 stratified bootstrap replicates.

Sensitivity of the results to the large proportion of negative samples was tested by repeating the analyses using a matching design, in which, from the 45,652 cattle, a *m:n* ratio of 1:4 was use, where for each positive test result of a given farm, four negative animals within the farm were selected as controls. The alternative matching design was tested for the four models (one global model and three age specific independent multivariable models).

The selection procedure of the final model in the matching study design was similar to that described above, with the exception that the random effect at the farm level was not incorporated because the matching adjusted for absence of independence.

All analyses were conducted using the R programming language version 3.3.2 [25]. Mixed models were fitted using the package lme4 [26] and the ROC analysis was performed using pROC [27].

## Results

### Descriptive results

Relatively few positive (*n* = 2865, 5.1%) and inconclusive (*n* = 470, 0.8%) test results were found among all 55,298 analyses performed during the study period. After exclusion of repeated tests and inclusive results, 45,652 assays were assessed, of which 2222 were positive (4.9%; CI_95%_: 4.6–6.0%). Samples originated from 691 herds, with a median of 30 animals samples per herd during the study period [interquartile range (IQR): 24–59]). Number of samples per month ranged from 1281 (October) to 5782 (November). Proportion of positive samples per month ranged from 3.0% (CI_95%_: 2.3–4.0%) to 6.1% (CI_95%_: 5.4–6.8%).

### Multivariable analysis

Initially, eleven variables were preselected based on a *p*-value of association ≤0.15 in the univariable step. In step two, the lactation number and milk fat content were excluded due to correlation with age (*r* = 0.93) and milk protein content (*r* = 0.53), respectively (Table 1). Similarly, 305ME was correlated with lactation persistence (*r* = 0.57) and excluded from further analysis.

**Table 1:** Estimated ratios along and coefficients with *p*-values of the fixed effects on factors associated with animal MAP-ELISA results from individual milk sample in 691 herds and 45,652 cows (tested ones) from four states in the north of USA

Variables	N	Positive (%)	Univariable analysis		Multivariable analysis			
			*p*-value*	odds ratio (CI_95%_)	β	S.E.	odds ratio (CI_95%_)	*p*-value*
Age (years)			< 0.001					< 0.001
1.25 to ≤4	26,152	3.5%	–	–	–	–	–	–
> 4 to ≤8	18,384	6.7%	< 0.001	2.20 (1.99–2.43)	0.724	0.053	1.99 (1.81–2.19)	< 0.001
> 8 to 15.29	1115	7.1%	< 0.001	2.43 (1.86–3.17)	0.728	0.137	1.98 (1.55–2.54)	< 0.001
Somatic cell count (×1000/ml)			< 0.001					< 0.001
1.0 to ≤ .4	11,711	3.2%	–	–	–	–	–	–
> 14 to ≤26	11,630	3.9%	0.05	1.15 (0.99–1.34)	0.058	0.078	1.05 (0.91–1.21)	0.45
> 26 to ≤39	10,622	5.3%	< 0.001	1.65 (1.43–1.91)	0.272	0.078	1.36 (1.18–1.56)	< 0.001
> 39 to 97	11,447	7.2%	< 0.001	2.33 (2.03–2.68)	0.467	0.077	1.66 (1.44–1.90)	< 0.001
Number of lactation			< 0.001					
0 to ≤1	14,072	3.3%	–	–				
> 1 to ≤2	15,521	4.4%	< 0.001	1.68 (1.47–1.92)				
> 2 to ≤3	8681	6.0%	< 0.001	2.36 (2.05–2.73)				
> 3 to 11	7378	7.4%	< 0.001	2.99 (2.58–3.46)				
Milk production (pounds)			< 0.001					< 0.001
0 to ≤52	12,220	7.7%	–	–	–	–	–	–
> 52 to ≤68	11,384	4.4%	< 0.001	0.53 (0.47–0.61)	− 0.507	0.066	0.66 (0.59–0.75)	< 0.001
> 68 to ≤86	11,465	3.7%	< 0.001	0.44 (0.38–0.51)	− 0.722	0.076	0.56 (0.49–0.65)	< 0.001
> 86 to 192	10,524	3.3%	< 0.001	0.35 (0.30–0.42)	−1.110	0.093	0.43 (0.36–0.51)	< 0.001
Milk fat (%)			0.03					
0 to ≤3.5	12,549	4.4%	–	–				
> 3.5 to ≤3.9	9903	4.5%	0.89	1.00 (0.88–1.15)				
> 3.9 to ≤4.5	12,657	4.7%	0.98	1.00 (0.87–1.13)				
> 4.5 to 9.9	10,543	5.9%	0.01	1.17 (1.03–1.34)				
Milk protein (%)			0.001					< 0.001
0 to ≤3.0	12,418	4.9%	–	–	–	–	–	–
> 3.0 to ≤3.3	13,952	4.1%	0.008	0.84 (0.73–0.95)	−0.262	0.072	0.76 (0.67–0.87)	< 0.001
> 3.3 to ≤3.5	8224	5.0%	0.61	0.96 (0.83–1.11)	− 0.261	0.083	0.74 (0.64–0.85)	0.001
> 3.5 to 7.4	11,058	5.7%	0.28	1.07 (0.93–1.23)	−0.380	0.083	0.67 (0.58–0.77)	< 0.001
Milk urea nitrogen (mg/dl)			0.17					
0 to ≤5	12,076	4.7%	–	–				
> 5 to ≤11	14,833	5.0%	0.77	1.02 (0.87–1.19)				
> 11 to ≤13	8087	5.0%	0.52	1.05 (0.88–1.26)				
> 13 to 81	10,656	4.7%	0.26	0.90 (0.76–1.07)				
Previous-305-d mature equivalent (305ME)(Kg)			< 0.001					
0 to ≤21,120	11,687	6.6%	–	–				
> 21,120 to ≤25,690	11,540	5.1%	< 0.001	0.69 (0.60–0.79)				
> 25,690 to ≤29,550	11,253	4.3%	< 0.001	0.55 (0.47–0.64)				
> 29,550 to 46,720	11,171	3.3%	< 0.001	0.42 (0.35–0.50)				
305-d mature equivalent (305ME)(Kg)			< 0.001					
0 to ≤20,220	11,718	6.8%	–	–				
> 20,220 to ≤25,370	11,485	5.1%	< 0.001	0.65 (0.57–0.74)				
> 25,370 to ≤29,400	11,256	4.2%	< 0.001	0.53 (0.46–0.62)				
> 29,400 to 46,720	11,192	3.2%	< 0.001	0.39 (0.33–0.46)				
Lactation persistence (%)			< 0.001					
1 to ≤100	14,022	6.1%	–	–				
> 100 to ≤108	9976	5.0%	< 0.001	0.79 (0.70–0.90)				
> 108 to ≤116	11,286	4.0%	< 0.001	0.60 (0.52–0.69)				
> 116 to ≤225	10,368	4.0%	< 0.001	0.58 (0.51–0.67)				
Days in milk			< 0.001					< 0.001
< 30	3658	6.4%	–	–	–	–	–	–
−31-60	3280	5.3%	0.39	0.90 (0.72–1.13)	0.007	0.120	1.00 (0.79–1.27)	0.94
61–90	1950	3.1%	< 0.001	0.49 (0.35–0.68)	−0.580	0.169	0.55 (0.40–0.78)	< 0.001
91–120	1604	4.5%	0.01	0.67 (0.48–0.92)	−0.283	0.167	0.75 (0.54–1.04)	0.08
121–150	1351	4.3%	0.01	0.63 (0.45–0.89)	−0.403	0.180	0.66 (0.46–0.95)	0.02
151–180	1224	5.6%	0.07	0.74 (0.53–1.03)	−0.243	0.173	0.78 (0.55–1.10)	0.16
181–210	1862	5.0%	0.22	0.83 (0.62–1.11)	−0.152	0.154	0.85 (0.63–1.16)	0.32
211–240	3336	3.6%	< 0.001	0.61 (0.46–0.80)	−0.513	0.143	0.59 (0.45–0.79)	< 0.001
241–270	6743	3.6%	< 0.001	0.57 (0.45–0.73)	−0.615	0.124	0.54 (0.42–0.69)	< 0.001
271–300	6498	4.6%	0.001	0.68 (0.54–0.85)	−0.558	0.124	0.57 (0.44–0.72)	< 0.001
> 301	14,146	5.7%	0.12	0.85 (0.69–1.04)	−0.557	0.116	0.57 (0.45–0.71)	< 0.001
Season of cow’s birth			0.07					
Winter (Dec-Feb)	12,339	5.3%	–	–				
Spring (Mar-May)	11,100	5.0%	0.16	0.91 (0.80–1.03)				
Summer (Jun-Aug)	12,134	4.5%	0.01	0.84 (0.74–0.96)				
Fall (Sep-Nov)	10,079	4.7%	0.05	0.88 (0.77–1.00)				

*p-values from the Likelihood-ratio test

Variables included in the final global mixed effect logistic model were age, SCC, milk production, milk protein, and DIM (Table 1). Continuous variables were used as categorized in the final multivariable models since they provided better fit to the data. Increasing age was associated with higher odds of seropositivity, especially in higher age classes (> 4 to ≤8 year-old; OR = 1.99; > 8 years OR = 1.98). Higher values of SCC were also associated with seropositive results (> 26 to ≤39; OR = 1.36 and values greater than 39; OR = 1.66). Milk production, percent of protein in milk and DIM were inversely associated with the risk of positive test results, so that animals with higher values in those variables had lower risk of being positive (Table 1).

The area under the ROC-curve for the final global model was 83.3% (CI_95%_: 82.5%–84.1%), indicative of a good predictive performance of the model. Fitted values from positive and negative observations were significantly different (Mann-Whitney test, *p*-value < 0.05).

Age-specific models were fitted separately, the model for both < 4 year-old and 4–8 year-old cattle included SCC, milk production, and DIM, while milk protein content was only selected in the model fitted on 4–8 year-old cattle (Additional file 1). Predictive power of both models was similar to the one determined in the general model, however the model fitted for young animals showed a better performance (AUC = 85.8 and 82.9 for young -- < 4 years, and adult cattle 4 ≤ 8 years old, respectively). In contrast, no variables were significantly associated with the test result for the older cattle (> 8 years).

Results from the matched design *m:n* were similar to those reported here, suggesting that the large proportion of negative animals did not affect the model outputs.

## Discussion

Although multiple studies have demonstrated the impact of MAP infection on production, there is much variability, in quantitative terms, in the results, which makes extrapolation of results to different settings difficult [6, 7, 10, 28]. The absence of official surveillance programs in the U.S. and standardized definitions for positive status to MAP further complicates the interpretation of epidemiological studies. The study here, which makes use of passive surveillance activities routinely and voluntarily performed by dairy herds, represents an attempt to identify the characteristics associated with seropositive animals in a high herd-prevalence setting. The small proportion of positive test results (4.9%) and large proportion of positive farms (55.4%; CI_95%_: 51.7–59.0%) is similar to figures obtained in previous studies, typically only a small portion of the animals in a herd test positive in serology-based results and hence individual seroprevalences are usually in the range of 3 to 10% [29–32], indeed other large serological studies perform outside U.S. reported occurrences of 2.3% and in a larger study MAP ranged from 5.6% in 2008, and 3.8% in 2015, peaking at 7% in 2009% both Denmark studies [33, 34], finally in China a 4.8% prevalence was reported using IDEXX ELISA [35].

Although SCC was here clearly associated with test result, with higher probability of positive results for those animals with increased counts (Table 1; Additional file 1), inconsistent results have been described in the literature [18, 36]. However, a strong association between somatic cells and antibodies against MAP has been found in Danish cows [37]. Somatic cell count was also shown to be strongly associated with higher risk for testing positive to MAP in 58,096 UK Holstein-Friesian cows, with higher SCC values in animals at higher and medium MAP risk compared with low-risk groups [12]. This effect was still evident once the possible influence of milk yield and age on probability of detection of MAP antibodies in milk was accounted for [12]. The SCC count could be useful when managing MAP, and should be interpreted altogether with other milk production parameters, such as milk yield and days in milk, that have been previously suggested to influence MAP milk ELISA results [11, 24].

The association between age of the tested animal and ELISA results (Table 1; Additional file 1) is similar to that described elsewhere [16] suggesting a limited ability of milk-ELISA for the discrimination of infected cows in young (< 3 year-old) animals. Age distribution in the tested population may therefore have a significant influence in the proportion of positive animals found. For example, some have found less than 0.33% < 2 year-old positive cattle, a proportion that increased to 0.94% for > 5 years-old animals [15]. For that reason, specific models were fitted for each age category. Although results were similar for < 4 and 4–8 year-old animals (Additional file 1), no associations were observed for > 8 year-old cattle, which may be explained, at least in part, by the reduced dataset (*n* = 1115), which may have affected the power to detect associations. The only difference between < 4 and 4–8 year-old cattle was the association of MAP milk ELISA result with milk protein content, which was not statistically significant for the younger cattle category, which may be due to limited impact of disease on production of animals in an early stage of MAP infection [6, 14].

Poorer performance has been observed in MAP-seropositive, compared to MAP-seronegative, animals [10]. Here, animals yielding > 86 pounds of milk per day were at a significantly lower risk of being positive (0.35, CI_95%_: 0.30–0.42) compared to the baseline category (Table 1). This could be due in part to a variation in the concentration of MAP-specific antibodies IgG in milk related with the milk production, so that animals with higher production experienced a dilution effect [11] Milk yield is also associated with age, but we observed the same association both in the multivariable model including age as a covariate and in the age-specific models, reinforcing the finding and suggesting an age-independent effect (Additional file 1). Although association was particularly strong for adult cattle, suggesting that older positive cows may have a more evident production decrease likely due to a more advanced stage of infection [6], young MAP-seropositive animals also show relatively poor performance, suggesting the effect of MAP infection was already significant at early stages of infection (Additional file 1). However, cows with higher milk yield are often under more stress, one should also consider the negative energy balance when analyzing animal performance and disease occurrences.

Days in milk associated negatively with MAP milk ELISA positive results especially after the first three months of lactation [11]. Our results from the global and the age-specific models are in agreement with these findings: both the global and the model fitted for the adult cows suggested that after 61 days in lactation the chances of finding a positive result decreased by almost half (Table 1, Additional file 1). Interestingly, in young cows this association was only identified after 211 days in milk, perhaps associated with a slower progress of infection at younger ages. This early negative association, for the global and adult cows model or later in the case of young animal, with days in milk is probably due to dilution of antibodies below the detection limit as suggested before [11]. The negative association between the number of liters produced and the sensitivity of MAP milk ELISA results should be further investigated. It would be interesting if data was collected in a higher resolution (i.e. weekly sampling instead of monthly), as this would allow one to capture smaller effects in the test result.

Higher protein content in milk was also associated with decreased odds of seropositivity as reported elsewhere [7]. Previously, this association was more evident for first lactation MAP-seropositive cows relative to cows that tested positive in later lactations [7], although other authors did not observe such association before 2 years of age [38]. Although an age-protein content interaction was not found in the model regardless of age, protein content was only identified as a significant covariate in the model for adult (4–8 year-old) age-specific model, suggesting a possible modulating effect of age on the association between MAP test result and protein content, at least for certain age classes.

No significant association between season of birth and odds of positivity for MAP was found here, in contrast with what has been previously described in 24 Jersey and 4 Holstein herds this may be related to different management strategies in the assessed populations [17]. Season temperatures in MN tend to fluctuate, according to MN department of natural resources Spring vary from 2 to 6 °C (north and south), Summer from 17 to 21 °C, Fall from 5 to 8 °C and Winter from − 13 to − 7 °C our model did not find an association as others [17] found a highest probability of infection during Summer months, such association should further be investigated especially in other countries with more favorable conditions of humidity and temperature.

A major limitation of the study here was the imperfect test accuracy of MAP milk ELISA, with the manufacturer indicating a test sensitivity at 0.52 and specificity at 0.98, which could lead to an underestimation of the prevalence and the existing associations in animals in earlier stages of infection. In addition, we only had access to dairy herds enrolled in the DHIA that were conducting MAP tests on some of their animals. These herds may have a higher level of awareness about the importance of the disease, which in turn could have result in different management strategies. However, the farms assessed here represent approximately 20% of all dairy herds in Minnesota (and 43% of all Minnesota DHIA herds) (Minnesota Department of Agriculture- https://www.mda.state.mn.us/food/business/~/media/Files/food/business/economics/dairyindprofile.pdf), but also representing a relatively good coverage for the Midwest region of the US. Finally, despite the limitations associated with the moderate correlation between results of milk and serum ELISA, milk samples are much more convenient, and more suitable to be use especially when considering large herds and countries with limited resources, where sampling is a big issue and to collect blood samples of such large population of cows for screening against MAP or any other disease is not possible.

## Conclusion

Although the proportion of positive serological results in our study population was low, MAP infection still impacting dairy herds health in the study region. Our results suggest that cows were more likely to test positive when they had SCC above 26 (× 1000/ml) – according to the global model the model for young (< 4 years) cows – and above 39 (× 1000/ml) based on the model fitted for older cows (4–8 years). Cows with higher milk production had a lower likelihood of testing positive in all models, as well as cows that were more than 61 days into their lactation (and over 211 days for cows < 4 years). Our results provide guidelines to help design MAP sampling strategies when production data is available, upon evidences of the models sampling animals with SCC between 26 and 39 (× 1000/ml), low milk production, that are in the initial stages of lactation from 61 to 211 days’ conditional on age would increase chances of identifying positive cows. However, results should be interpreted with care given the synergistic effect of several variables (age, milk production, days in milk), and the possible impact that other physiological and clinical condition (e.g. mastitis) may have, especially on the SCC counts.

## Additional file

Additional file 1: Estimated ratios and coefficients with *p*-values of the fixed effects on factors associated with animal MAP-ELISA results from individual milk sample for young cows (26,153 animals), adult cows (18,384 animals) from four states in the north of USA. (DOCX 21 kb)

## Abbreviations

305ME: 305-day mature equivalent milk production
DHIA: Dairy Herd Improvement Association
DIM: days in milk
ELISA: Enzyme Linked Immunosorbent Assay
IQR: interquartile range
MAP: Mycobacterium avium subsp. paratuberculosis
OR: odds ratios
ROC: receiver operating characteristic
SCC: somatic cell count
