# Supplementary material for: Identifying individual animal factors associated with Mycobacterium avium subsp. paratuberculosis (MAP) milk ELISA positivity in dairy cattle in the Midwest region of the United States
Source: BMC Vet Res. 2018 Jan 25;14:28. doi: 10.1186/s12917-018-1354-y (PMC5784586; doi:10.1186/s12917-018-1354-y)
Supplement: Additional file 1: — Estimated ratios and coefficients with p-values of the fixed effects on factors associated with animal MAP-ELISA results from individual milk sample for young cows (26,153 animals), adult cows (18,384 animals) from four states in the north of USA. (DOCX 21 kb) [file 12917_2018_1354_MOESM1_ESM.docx]

**Additional file 1 -** Estimated ratios and coefficients with p-values of the fixed effects on factors associated with animal MAP-ELISA results from individual milk sample for heifers cows (26,153 animals), adult cows (18,384 animals) from four states in the north of USA.

| Variables | N | Positive (%) | Multivariable analysis | |
| --- | --- | --- | --- | --- |
|  |  |  | p-value* | odds ratio (CI_95%_) |
| Young cows (<4 years) |  |  |  |  |
| Somatic cell count (x1000/ml) |  |  | <0.001 |  |
| 1.0 to ≤ 14 | 8486 | 2.5% | - | - |
| >14 to ≤ 26 | 7271 | 3.0% | 0.77 | 1.03 (0.83-1.26) |
| >26 to ≤ 39 | 5571 | 4.1% | 0.003 | 1.37 (1.11-1.70) |
| >39 to 97 | 4694 | 5.4% | <0.001 | 1.76 (1.42-2.19) |
|  |  |  |  |  |
| Milk production (pounds) |  |  | <0.001 |  |
| 0 to ≤ 52 | 5851 | 5.9% | - | - |
| >52 to ≤ 68 | 7100 | 3.3% | <0.001 | 0.65 (0.53-0.80) |
| >68 to ≤ 86 | 7457 | 2.7% | <0.001 | 0.58 (0.46-0.74) |
| >86 to 192 | 5701 | 2.1% | <0.001 | 0.46 (0.34-0.63) |
|  |  |  |  |  |
| Days in milk  <30  31-60  61-90  91-120  121-150  151-180  181-210  211-240  241-270  271-300  >301 | 2361  2022  1109  949  660  649  1066  2133  4353  3936  6915 | 5.6%  3.6%  3.2%  3.6%  3.6%  5.9%  3.2%  2.2%  2.4%  3.3%  3.8% | -  0.60  0.38  0.56  0.58  0.65  0.56  0.007  <0.001  0.009  0.02 | -  0.91 (0.65 1.28)  0.82 (0.52-1.27)  0.87 (0.54-1.39)  0.86 (0.51-1.46)  1.11 (0.69-1.76)  0.87 (0.54-1.38)  0.55 (0.36-0.85)  0.53 (0.37-0.77)  0.61 (0.42-0.88)  0.66 (0.47-0.94) |
|  |  |  |  |  |
| Adult cows (4-8 years) |  |  |  |  |
| Somatic cell count (x1000/ml) |  |  | <0.001 |  |
| 1.0 to ≤ 14 | 3109 | 5.0% | - | - |
| >14 to ≤ 26 | 4173 | 5.3% | 0.82 | 1.02 (0.80-1.30) |
| >26 to ≤ 39 | 4756 | 6.6% | 0.16 | 1.18 (0.93-1.49) |
| >39 to 97 | 6246 | 8.9% | 0.001 | 1.43 (1.44-1.79) |
|  |  |  |  |  |
| Milk production (pounds) |  |  | <0.001 |  |
| 0 to ≤ 52 | 5914 | 9.3% | - | - |
| >52 to ≤ 68 | 4027 | 6.0% | <0.001 | 0.60 (0.50-0.72) |
| >68 to ≤ 86 | 3804 | 5.6% | <0.001 | 0.49 (0.40-0.61) |
| >86 to 192 | 4624 | 4.7% | <0.001 | 0.32 (0.25-0.41) |
|  |  |  |  |  |
| Milk protein (%) |  |  | <0.001 |  |
| 0 to ≤ 3.0 | 4417 | 7.1% | - | - |
| >3.0 to ≤ 3.3 | 5133 | 5.7% | <0.001 | 0.71 (0.58-0.86) |
| >3.3 to ≤ 3.5 | 3387 | 7.0% | 0.01 | 0.76 (0.60-0.95) |
| >3.5 to 7.4 | 5447 | 7.0% | <0.001 | 0.60 (0.48-0.75) |
|  |  |  |  |  |
| Days in milk |  |  | <0.001 |  |
| <30 | 1228 | 7.7% | - | - |
| 31-60 | 1165 | 8.1% | 0.49 | 1.13 (0.79-1.60) |
| 61-90 | 788 | 3.2% | <0.001 | 0.39 (0.23-0.65) |
| 91-120 | 616 | 5.7% | 0.08 | 0.65 (0.40-1.05) |
| 121-150 | 637 | 5.0% | 0.02 | 0.57 (0.34-0.94) |
| 151-180 | 533 | 5.4% | 0.03 | 0.57 (0.34-0.97) |
| 181-210 | 749 | 7.3% | 0.51 | 0.86 (0.56-1.33) |
| 211-240 | 1152 | 6.2% | 0.06 | 0.68 (0.45-1.02) |
| 241-270 | 2282 | 5.5% | 0.004 | 0.58 (0.40-0.84) |
| 271-300 | 2448 | 6.3% | 0.004 | 0.59 (0.41-0.85) |
| >301 | 6786 | 7.5% | 0.005 | 0.61 (0.44-0.86) |

*p-values from the Likelihood-ratio test
